# Determinants, reasons for choice and willingness to recommend birthing facility among mothers in public and private health facilities in Ebonyi, Nigeria

**DOI:** 10.11604/pamj.2021.38.289.24437

**Published:** 2021-03-19

**Authors:** Ijeoma Nkem Okedo-Alex, Ifeyinwa Chizoba Akamike, Johnbosco Ifunanya Nwafor, Chika Nwakanma Onwasigwe

**Affiliations:** 1 Department of Community Medicine, Alex Ekwueme Federal University Teaching Hospital Abakaliki, Ebonyi State, Nigeria,; 2 Department of Obstetrics and Gynaecology, Alex Ekwueme Federal University Teaching Hospital Abakaliki, Ebonyi State, Nigeria

**Keywords:** Facility-based, childbirth, skilled birth, attendance, public hospital, private hospital, determinants, maternal health, Nigeria

## Abstract

**Introduction:**

facility-based births remain low in Nigeria despite the enormous benefits on maternal and neonatal health. We compared the determinants, reasons for choice and willingness to recommend public and private birthing facilities among mothers in Ebonyi, Nigeria.

**Methods:**

this was a cross-sectional survey among 620 women whose childbirth occurred in public (teaching) and private-for-profit mission hospitals in Ebonyi, Nigeria. Semi-structured, interviewer-administered questionnaires were used for data collection.

**Results:**

the mean age of the respondents was 29.86±4.4. Most had post-secondary education (71.0%), more than 4 antenatal visits (83.4%) and vaginal births (77.7%). Respondents with high economic status [adjusted odds ratio (aOR) 2.88; Confidence Interval (CI) 1.98-4.18], post-secondary education (aOR 1.73; CI 1.13-2.64) and urban residence (aOR 3.51; CI 2.19-5.61) were more likely to utilize public birthing facilities. In the private hospital, religion (78.4%) was the commonest reason for utilization while poor quality of services (61.9%) was the major cause of dissatisfaction. In the public hospital, the main reason for patronage was insurance enrolment (73.2%) while negative provider attitude (66.7%) led to dissatisfaction. In both facilities, majority (92%) were willing to recommend their birth facility to others.

**Conclusion:**

regardless of facility type, respondents were willing to recommend or reuse the health facility for subsequent obstetric care. Religion and insurance enrolment were the major reasons for choosing the private and public hospital respectively. Residence, educational and income status influenced birthing facility type used. We recommend improved quality of services in private hospitals and provision of insurance with improved provider attitude in public health facilities.

## Introduction

Although the global maternal mortality rate (MMR) declined by 44% since 1990, it fell well short of the targeted 75% reduction as stated in the 5^th^ millennium development goal (MDG). The World Health Organization (WHO) African region made the least progress in reducing MMR and accounted for 64% of maternal deaths in 2015 [1]. Globally, out of the 135 million live births that occurred in 2011, about 46 million of the women delivered alone or with inadequate care [2]. In the Nigerian health system, 26% of deliveries occur in public sector facilities, and 13% occur in private sector facilities making up the national 39% skilled birth attendance [3]. Given the benefits of skilled birth attendance and the attendant potential complications that can arise during child birth, it is worrisome that about 61% of Nigerian women still deliver at home. This undoubtedly contributes to the high burden of maternal mortality in the country [3-5]. In Ebonyi State, less than 60% of women give birth in health facilities and are attended to by a skilled birth attendant. The state has the lowest rates of facility-based childbirth and skilled birth attendance among the five states in south-Eastern Nigeria with about 40% of women delivering their babies at home [6,7]. Maternal age, educational status, place of residence, employment status, parity, marital status, autonomy in decision making and socio-economic status have been found to influence utilization of health facilities for childbirth [8-10]. Other factors that influence birthing in both health facilities and preference for home births include cost of services, geographical access, physical comfort, staff attitude, fear of mistreatment, quality services, antenatal attendance, complications during labour, dissatisfaction with previous treatment received at the health facility [11-15]. Women want and expect quality maternal care that promotes wellbeing for mothers and their babies. These expectations influence the choice of where to have their babies [16]. This study compared the determinants, reasons for choice of birth place and willingness to recommend birth facility among mothers whose childbirth occurred in public and private health care settings in Southeast Nigeria.

## Methods

**Study setting:** the study was conducted in Afikpo and Abakaliki, the major cities of Ebonyi State in the South-eastern part of Nigeria. According to the 2006 population and housing census, the population of Ebonyi State is approximately 2,176,947 with an area of 5,935 square kilometres [17]. There are 3 senatorial zones and 13 Local Government Areas (LGAs) in the State. The majority of Ebonyi people are Ibos and farmers by occupation. Ebonyi State has 2 tertiary health facilities, 13 general hospitals, 534 primary health centres, and 6 faith-based (mission) hospitals. Over 50% of health services in the state are provided by the mission hospitals which mostly operate on a private-for-public basis [7]. This study was conducted in the only teaching hospital (Alex Ekwueme Federal University Teaching Hospital) Abakaliki and Mater Misericodiae catholic mission, Afikpo both in Ebonyi State.

**Study design and population:** this study was a comparative cross-sectional study among mothers whose childbirths occurred in the selected facilities. They were recruited through the immunization clinics of the health facilities. Women who were more than fourteen (14) weeks after childbirth were excluded from the study.

**Sample size and sampling technique:** using the sample size formula for comparing two proportions with P1 and P2 of 98% [18] and 93% respectively at a desired power of 80% and a significance level of 5%, the calculated sample size was 269. Using an attrition rate of 20%, the sample size was then recalculated as 294 per facility. In each facility, 310 participants were then recruited. The immunization clinic in the private hospital holds once every week with an average client load of 100 per immunization day. In the public health facility, the immunization clinics are held twice weekly and have about 80 clients per immunization clinic day. Systematic random sampling was used to select the respondents using the immunization attendance registers. The sampling interval (K) was calculated by dividing the average number of immunization clinic attendees by the number of study participants to be recruited that day. The sampling interval in the private hospital was 4 while that in the public hospital was 6.

**Data collection:** data were collected using semi-structured interviewer-administered questionnaires. The questionnaire collected information on socio-demographic and other characteristics of the respondents, reasons for choice of birthing facility, satisfaction and dissatisfaction with facility quality of care attributes and willingness to reuse or recommend the health facility for future obstetric needs.

**Data analysis:** the Statistical Package for Social Sciences (SPSS) for Microsoft Window version 20 software was used for entry and analysis of the data. The STATA statistical software version 12 [19] was used to develop the socioeconomic status index using principal component analysis (PCA). Proportions, means and standard deviations were calculated for the appropriate variables. The association between the place of childbirth and the independent variables was assessed using Chi-square test statistic. Both univariable and multivariable regression analyses were performed to assess factors associated with utilization of public or missionary birthing facility. The p-value of 0.2 on Chi square (univariable) analyses [20] was used as a cut-off for inclusion of variables modelled in binary logistic regression to isolate correlates of the dependent variable at 5% level of significance. The correlates were presented using confidence intervals (CI), crude odds ratio (cOR) and adjusted odds ratio (aOR).

**Ethical approval:** ethical approval for this study was secured from the research and ethics committee of Alex Ekwueme Federal University Teaching Hospital, Abakaliki, Ebonyi State. Participants provided written informed consent after being informed of the purpose of the study, their rights and responsibilities as participants. They were assured of their voluntary participation and confidentiality of their responses.

## Results

**Socio-demographic characteristics of the respondents:** the mean age of the respondents was 29.86±4.4. Most had post-secondary education (n=440, 71.0%), were employed (n=522, 84.2%) and resided in the urban area (n=466, 75.2%). Majority of the women was multiparous (n=440, 71.0%), had at least 4 antenatal visits (n=517, 83.4%) and used the same facility for childbirth facility and antenatal care in their immediate past confinement (n=574, 92.6%) (Table 1).

**Table 1:** socio-demographic and other characteristics of the respondents

Variable	Frequency	Percentage (%)
**Age (years)**		
**<**30	303	48.9
≥30	317	51.1
**Mean age (mean ±SD)**	29.86±4.4	
**Marital status**		
Currently unmarried^	35	5.6
Currently married	585	94.4
**Educational level**		
Secondary and less	180	29.0
Post-secondary	440	71.0
**Religious denomination**		
Catholic	298	48.1
Others+	322	51.9
**Employment status**		
Unemployed	98	15.8
Employed	522	84.2
**Place of residence**		
Rural	154	24.8
Urban	466	75.2
**Socio-economic status**		
Low socio-economic status	396	63.9
High socio-economic status	224	36.1
**Parity**		
Primipara	180	29.0
Multipara	440	71.0
**Mode of childbirth**		
Vaginal delivery	482	77.7
Caesarean Section	138	22.3
**Time of childbirth**		
Daytime	346	55.8
Night time	274	44.2
**HIV status**		
Negative	549	88.5
Others^	71	11.5
**Decision on childbirth facility**		
Individual-based	251	40.5
Joint as couple	369	59.5
**Number of antenatal visits**		
3 and less	103	16.6
≥4	517	83.4
**ANC facility same as childbirth facility**		
No	46	7.4
Yes	574	92.6
**Type of skilled birth attendant**		
Doctor	396	63.9
Nurse/Midwife	224	36.1

**Reasons for choice of birthing facility:** religion was the most common reason for choosing the private hospital as a birth facility (n=40, 78.4%) while other reasons (mostly NHIS enrolment) was the most common reason given by respondents in the teaching hospital (n=41, 73.2%). Other commonly cited reasons by respondents in the private hospital were being referred (n=37, 72.5% vs. n=14, 27.5%; p=0.001) and partner´s choice (n=106, 67.5% vs. n=51, 32.5%; p=0.001). In the teaching hospital, being booked in the facility (n=103, 58.9% vs. n=72, 41.1%; 0.006) and quality of services (n=199, 51.0% vs. n=191, 49.0%; p=0.506) were the other commonly cited reasons for choosing the teaching hospital as childbirth facility (Table 2).

**Table 2:** reasons for choice of birthing facility given by the respondents

Variable	Private hospital n=310 Yes (%)	Public hospital n=310 Yes (%)	p value
Reduced cost of services	84(66.1)	43(33.9)	<0.001
Short waiting time	64(67.4)	31(32.6)	<0.001
Proximity/accessibility	114(63.3)	69(37.7)	<0.001
Quality of services	191(49.0)	199(51.0)	0.506
Provider attitude	116(64.4)	64(35.6)	<0.001
Provider expertise/equipment	4(66.7)	2(33.3)	0.412
Partner's choice	106(67.5)	51(32.5)	<0.001
No particular reason	30(61.2)	19(38.8)	0.102
Conducive childbirth environment	134(61.8)	83(38.2)	<0.001
Previous experience	91(58.3)	65(41.7)	0.016
Recommended to me	66(64.7)	36(35.3)	0.001
Referred	37(72.5)	14(27.5)	0.001
Religion	40(78.4)	11(21.6)	<0.001
Booked here	72(41.1)	103(58.9)	0.006
National health insurance scheme (NHIS) enrolment	15(26.8)	41(73.2)	<0.001

**Satisfaction and dissatisfaction with delivery facility quality of care attributes:** more respondents in the private hospital than those in the public hospital were satisfied with the following: reduced cost of services (n=91, 63.9% vs. n=52, 36.4%; p<0.001), short waiting time (n=87, 69% vs. n=39, 31.0%; p<0.001), proximity (n=121, 61.1% vs. n=77, 38.9%; p<0.001), good provider attitude (n=164, 52.6% vs. n=134, 43.2%; p=0.016), conducive childbirth environment (n=174, 52.2% vs. n=141, 44.8%; p=0.001) and patient friendly programs (n=98, 63.2% vs. n=57, 36.8%; p=0.008) (Table 3). A higher proportion of respondents in the public hospital were dissatisfied with: high cost of services (n=105, 57.7% vs. n=77, 42.3%; p=0.014), and negative provider attitude (n=38, 66.7% vs. n=19, 33.3%; p=0.008)). More respondents in the private hospital were not dissatisfied with any quality of care attribute in the facility (n=112, 59.3% vs. n=105, 40.7%; p=0.002) (Table 3).

**Table 3:** satisfaction and dissatisfaction with delivery facility quality of care attributes among respondents in the private and public hospitals

Variable	Private hospital n=310 Yes (%)	Public hospital n=310 Yes (%)	p value
**Satisfaction with birthing facility quality of care attributes**			
Quality of services	207 (48.8)	217 (51.2)	0.388
Conducive childbirth environment	174 (52.2)	141 (44.8)	0.008
Good provider attitude	164 (52.9)	134 (43.2)	0.016
Proximity/accessibility	121 (61.1)	77 (38.9)	<0.001
Patient-friendly programs	98 (63.2)	57 (36.8)	<0.001
Reduced cost of services	91 (63.6)	52 (36.4)	<0.001
Short waiting time	87 (69.0)	39 (31.0)	<0.001
Others	19 (48.7)	20 (51.3)	0.869
**Dissatisfaction with birthing facility quality of care attributes**			
None as I don't dislike anything about the facility	112 (59.3)	77 (40.7)	0.002
High cost of services	77 (42.3)	105 (57.7)	0.014
Long waiting time	38 (44.2)	48 (55.8)	0.245
Far distance from me	19 (61.3)	12 (38.7)	0.197
Negative provider attitude	19 (33.3)	38 (66.7)	0.008
Lack of patient-friendly programs	16(51.6)	15 (48.4)	0.854
Others	15 (32.6)	31 (67.4)	0.014
Poor quality of services	13 (61.9)	8 (38.1)	0.267
Unconducive childbirth environment	11 (35.5)	20 (64.5)	0.097

**Correlates of choice of birthing facility:** the variables that were statistically significant p-value of <0.2 on univariable analysis were socioeconomic status, number of ANC visit's marital status, place of residence status HIV status, decision-maker on childbirth facility ANC facility same as childbirth facility. Those with high socioeconomic status were 2.88 times more likely to utilize the public hospital for childbirth than those with low socioeconomic status (CI 1.98-4.18). Urban residents were 3.51 times more likely to use public birthing facility compared to the rural counterparts (CI 2.19-5.61). Respondents with post-secondary school education were 1.73 times more likely to have their babies in the public health facility compared to those who had less than secondary school education (CI 1.13-2.64) (Table 4).

**Table 4:** univariable and multivariable correlates of choice of birthing facility among the respondents

Variable	Univariable analysis		Multivariable analysis	
	OR (95% CI)	P value	OR (95% CI)	P value
**Socio-economic status**				
High socio-economic status	4.75 (3.38-6.67)	<0.001	2.88(1.98-4.18)	<0.001
Low socio-economic status	1		1	
**Age (years)**				
≥30	1.28 (0.93-1.75)	0.271	1.06 (0.741-1.52)	0.757
<30	1		1	
**Number of ANC visits**				
>4	3.21 (2.01-5.13)	<0.001	1.59 (0.93-2.71)	0.092
3 and less	1		1	
**Marital status**				
Currently married	3.60 (1.61-8.06)	0.002	2.10 (0.85-5.22)	1.110
Currently unmarried^	1		1	
**Place of residence**				
Urban	5.92 (3.83-9.15)	<0.001	3.51 (2.19-5.61)	<0.001
Rural	1		1	
**HIV status**				
Others^	0.41 (0.24-0.69)	0.001	0.69 (0.38-1.28)	0.247
Negative	1		1	
**Decision-maker on childbirth facility**				
Joint as couple	2.11 (1.52-2.92)	<0.001	1.37 (0.94-2.00)	0.102
Individual-based	1		1	
**ANC facility same as childbirth facility**				
Yes	1.97 (1.05-3.69)	0.035	1.16 (0.57-2.39)	0.681
No	1		1	
**Educational level**				
Post-secondary	3.02 (2.09-4.37)	<0.001	1.73 (1.13-2.64)	0.012
Secondary and less	1		1	

**Figure 1:**
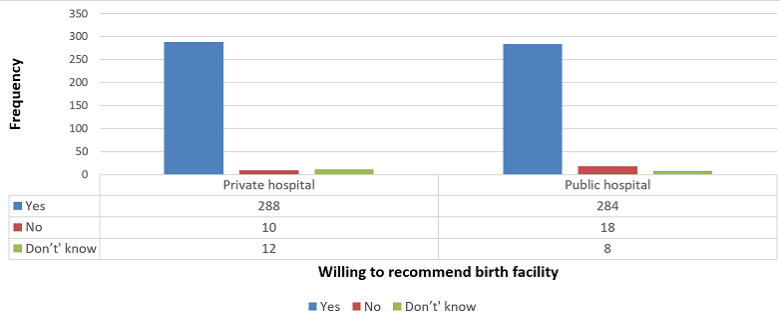
willingness of respondents to recommend birth facility

**Willingness to recommend childbirth facility:** majority of respondents in both hospitals were willing to recommend their birth facility to others. This proportion was slightly higher in the private hospital however this difference was not statistically significant (private hospital= 92.9% vs public hospital= 91.6%, P=0.211 (Figure 1).

## Discussion

We compared the determinants, reasons for choice of birth place and willingness to recommend birth facility among mothers whose childbirth occurred in public and private health care settings in Ebonyi, Nigeria. We found that poverty, low educational status and rural residence made women less likely to utilize the public hospital as a birthing facility. Poverty and low educational status (which are mostly found in the rural setting) could make mothers unaware of the clinical services available in such a public health facility as a teaching hospital. This could also influence their perceived costs of services in the public health facility. Although the mission hospital was for profit, poor women may have received subsidized or free maternal health care based on humanitarian grounds. Also, public health facilities have been associated with reduced quality of care manifested in unfriendly staff attitude, disrespectful care, stressful hospital protocols, poor accountability mechanisms, inadequate supplies and equipment amongst others [21-24]. Additionally, women may be inclined to think that there will be better outcomes in a mission hospital probably because they feel that God is present there and that health workers will carry out their duties with the fear of God considering their work as a vocational calling [25]. This is especially so in Nigeria where most pregnant women believe that pregnancy and childbirth have spiritual underpinnings and so tend to look for where they can get both spiritual and medical attention [26]. Women who belonged to the higher socioeconomic class have also been shown to prefer private maternity care services [27,28]. In contrast, other studies have found that women from lower educational status tended to have their babies in public health facility than in private hospitals [23,29]. The respondents in both facilities expressed various reasons for their choice of birth facilty. The commonly cited reasons among respondents in the private hospital were religion, being referred and partner´s choice. This could be explained by the socio-demographic data as almost half of the respondents in the mission hospital were of the Catholic denomination and jointly decided with their spouse to deliver at the mission hospital. Respondents who delivered in the teaching hospital chose to do so because of other reasons such as insurance services in the hospital, being booked as antenatal clients and quality of services. Antenatal booking and attendance and desire for quality maternal health services has also been cited as reasons for choosing institutional child birth in other studies [15,30].

The preferred quality of care attributes of the mission hospital were the short waiting time, reduced cost of services and patient-friendly programs available in the facility. This largely corroborates with findings from other studies [29]. In contrast, the respondents who had delivered in the teaching hospital cited quality of services, good provider attitude and conducive birth environment as the major quality of care attributes that were satisfactory about the teaching hospital as a birth facility. This is different from the findings of another study that reported low levels of satisfaction with provider attitude and interaction in public hospitals [31]. Similar to the preferences expressed by respondents in the mission hospital, poor quality of services was the most unsatisfactory attribute by the respondents while those in the teaching hospital opined that other reasons such as hospital protocols made quality of care at the teaching hospital unsatisfactory. Long waiting time and poor quality of maternal health services have been found to act as deterrents to the utilisation of obstetric services in health facilities [11,12]. It is important for health facilities to sustain the identified features that promote satisfaction with institutional childbirth while on the other hand mitigating the unsatisfactory qualities cited by these women in order to promote skilled care utilisation and reduce maternal mortality. This is especially so as women who are dissatisfied with previous obstetric care given to them are less likely to return to utilise such care for subsequent pregnancies. Such dissatisfied women can also discourage other women from institutional child birth [13].

The majority of the women in both facilities (91%) were willing to recommend the facility to other women. This high level of willingness to recommend the birth facility in spite of the quality of care concerns highlighted may suggest limited options for specialist obstetric care in the State. Likewise, a similar study among Nigerian women in Benue State found that although the women in the study experienced various forms of mistreatment during childbirth, most of them agreed that these experiences would not discourage their intended use of health facilities. This was adduced to be due to their perceived inherent lack of choice (of place of delivery given the alternatives available) and an underlying sense of helplessness (being at the mercy of health providers) [31]. However, the willingness of these women to recommend and use the birth facilities in the future should serve as incentive for improving the quality of maternal health care for parturients. Other studies have also reported high levels of willingness to recommend birth facility by respondents [32]. Some limitations of this study are firstly, the inherent limitation of a cross-sectional study design does not permit causal relationship inferences or establish temporality. Second, because the findings were based on self-report and recall, the study could be prone to social desirability and recall biases. The study has limited external validity because it was conducted in a restricted number of facilities in Southeast Nigeria.

## Conclusion

In this study, there was a high level of willingness to reuse or recommend the health facility for subsequent obstetric care. In the private hospital, religion and being referred were the most common reasons for choosing the mission hospital while other reasons such as enrolment in health insurance, operational convenience because self or spouse works in the hospital were the most common reasons for choosing the teaching hospital as a childbirth facility. High educational status, economic status and urban residence made women more likely to utilize the public hospital as a birthing facility. We recommend improved quality of services in private hospitals and provision of insurance with improved provider attitude in public health facilities.

### What is known about this topic

- Facility-based childbirth rates are sub-optimal in Nigeria and other developing countries;
- Public health facilities are often associated with poor quality of care in developing countries.

### What this study adds

- Regardless of facility type, willingness to recommend or reuse birthing facility for subsequent obstetric care was high;
- Religion, being referred and partner’s choice were reasons for choice of private hospitals as against insurance services and quality of services in public health facilities;
- Poor quality of services of services and negative provider attitude were the major sources of dissatisfaction in the private and public hospital respectively.
